# Is the glucocorticoid receptor a key player in prostate cancer?: A literature review

**DOI:** 10.1097/MD.0000000000029716

**Published:** 2022-07-22

**Authors:** Minas Sakellakis, Laura Jacqueline Flores

**Affiliations:** a Department of Genitourinary Oncology, MD Anderson Cancer Center, University of Texas, Houston, Texas, United States; b Department of Stem Cell Transplantation and Cellular Therapy, MD Anderson Cancer Center, University of Texas, Houston, Texas, United States.

**Keywords:** androgen, cancer, glucocorticoid, prostate, receptor, signaling

## Abstract

Glucocorticoids act through the glucocorticoid receptor (GR) and exert pleiotropic effects in different cancer types. In prostate cancer cells, GR and androgen receptor (AR) share overlapping transcriptomes and cistromes. Under enzalutamide treatment, GR signaling can bypass AR activation and promote castration resistance via the expression of a subset of AR-target genes. However, GR-dependent growth under enhanced antiandrogen inhibition occurs only in a subset of primed cells. On the other hand, glucocorticoids have been used successfully in the treatment of prostate cancer for many years. In the context of AR signaling, GR competes with AR for DNA-binding and has the potential to halt the proliferation rate of prostate cancer cells. Their target genes overlap by <50% and they execute unique functions in vivo. In addition, even when AR and GR upregulate the same transcriptional target gene, the effect might not be identical in magnitude. Besides being able to drive tumor proliferation, GR is also a key player in prostate cancer cell survival. Stimulation of GR activity can undermine the effects of enhanced antiandrogen treatment, chemotherapy and radiotherapy. GR activation in prostate cancer can increase prosurvival gene expression. Identifying the full spectrum of GR activity will inform the optimal use of glucocorticosteroids in prostate cancer. It will also determine the best strategies to target the protumorigenic effects of GR.

## 1. Introduction

Prostate cancer is one of the leading causes of cancer-related death worldwide.^[1]^ The disease is primarily driven by androgen receptor (AR) activity. Suppression of AR signaling through pharmaceutical or surgical castration, along with the use of newer generation antiandrogen therapy (e.g., abiraterone acetate, enzalutamide, apalutamide, darolutamide), is the mainstay of treatment for androgen-sensitive prostate cancer.^[2]^ Acquired castration resistance occurs through the restoration of AR signaling via AR amplification, AR mutation, and aberrant AR coregulator activity.^[2]^ Prostate cancer can also escape castration therapy via intratumoral androgen biosynthesis.^[2]^ AR signaling can also be reactivated through the expression of constitutively active AR splice variants (such as androgen receptor splice variant 7), which lack the ligand-binding domain of AR, but contain the amino-terminal and the DNA-binding domain.^[2]^ Ligand-independent AR activation can also be achieved through the p100/p52 pathway.^[3]^ Aberrant activation of the phosphoinositide 3-kinase/AKT pathway, which is a downstream pathway of several key receptor tyrosine kinases, such as c-met, insulin-like growth factor receptor, and epidermal growth factor receptor, has also been associated with the development of castration-resistant prostate cancer (CRPC).^[3,4]^ Several receptor tyrosine kinases, including insulin-like growth factor-1 receptor and epidermal growth factor receptor, have been shown to enhance AR stability and activity.^[3,5,6]^ Human epidermal growth factor receptor 2 (HER2/neu) activity can also phosphorylate AR, resulting in AR activation.^[6]^ In addition, antiandrogen therapy can result in upregulation of the glucocorticoid receptor (GR), which can bypass AR activation and promote castration resistance via direct expression of a subset of AR-target genes.^[7]^ Later stages of the disease are characterized by growth that is completely independent of AR signaling.^[8]^ This review focuses on the importance of GR in the regulation of disease progression and resistance to antiandrogen therapy.

## 2. Methods

An electronic search of the PubMed and Google Scholar databases was performed. We carefully examined data from the most relevant literature, with respect to the structure and function of the GR in prostate cancer. We emphasized GR activity during the early stages of the disease, particularly in relation to AR activity and AR inhibition. We also searched for GR activity in other tissues or tumor types that might be useful in research regarding the role of the GR in prostate cancer. This is a review article, thus ethical approval was not necessary.

## 3. Discussion

### 3.1. The GR structure and function

**Figure 1.**
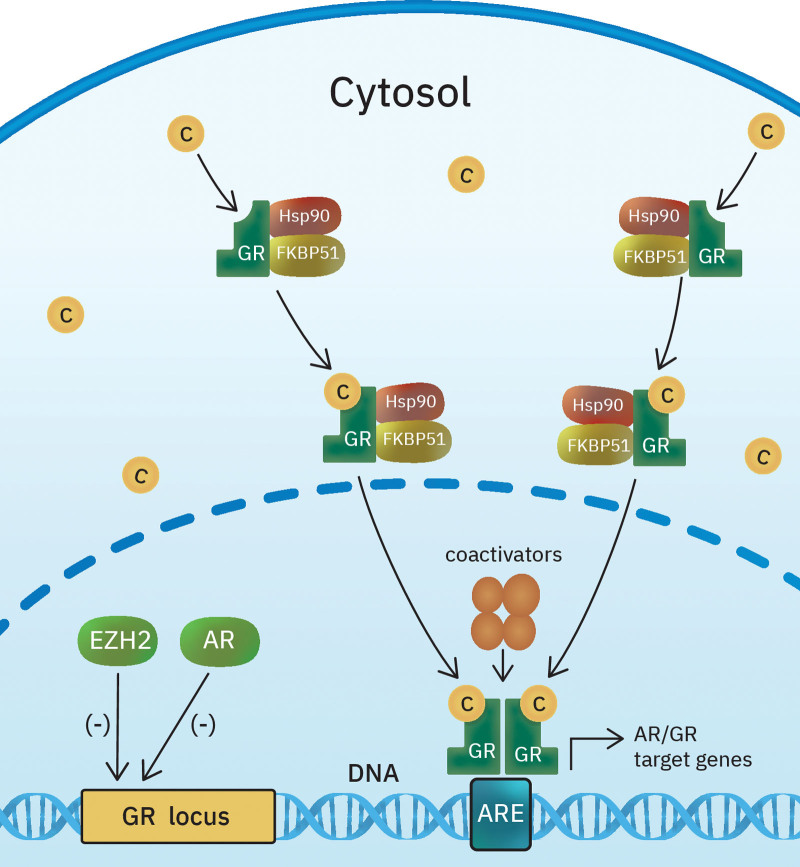
Schematic of GR gene expression regulation and GR transactivation/transrepression in prostate cancer. In prostate cancer, GR expression is initially silenced by EZH2-mediated repression and AR binding at the GR locus. However, in subsets of castration-resistant tumors, GR expression is restored by reversion of both repressive signals. Unliganded GR resides in the cytosol, where it is complexed with proteins such as Hsp90 and FK506 immunophilins. The c-bound receptor undergoes conformational changes and translocates into the nucleus. GR and AR share overlapping transcriptomes and cistromes, and they compete for DNA binding. GR is the direct driver of a subset of critical AR-target genes. AR = androgen receptor, ARE = androgen response element, c = cortisol, EZH2 = enhancer of zeste 2 polycomb repressive complex 2 subunit, FK506 = tacrolimus, GR = glucocorticoid receptor, Hsp90 = heat shock protein 90.

GR is a member of the nuclear receptor superfamily of intracellular receptors. The human gene encoding GR (*nuclear receptor subfamily 3 group C member 1*) is located on chromosome 5.^[9]^ The GR gene promoters contain binding sites for several transcription factors (e.g., activator protein-1, interferon-regulatory factor) and GR itself, thereby regulating its own expression.^[10–13]^ GR promoters are also subject to epigenetic regulation.^[10,14]^ The GR protein consists of 3 functional domains: the N-terminal domain, DNA-binding domain, and C-terminal ligand-binding domain.^[9]^ N-terminal domain houses constitutively active transcriptional activation factor 1.^[10]^ Ligand-dependent transcriptional activation factor 2 is located in the ligand-binding domain. Both activation factor 1 and activation factor 2 are critical for the interaction of GR with tissue-specific coactivators.^[10,15]^ GR is the principal receptor responsible for the physiological and pharmacological effects of cortisol and other glucocorticoids in almost every cell in the body.^[10]^ Unliganded GR resides in the cytosol, where it is complexed with various proteins such as chaperones (e.g., p23, heat shock protein [Hsp]70, and Hsp90) and immunophilins of the tacrolimus (FK506) family (e.g., FK506-binding protein 51, FK506-binding protein 52).^[16]^ These proteins maintain the GR in an inactive conformation.^[10,16]^ However, glucocorticoid hormones can diffuse through the cell membrane into the cytosol and bind to GRs with high affinity (Fig. 1). The ligand-bound receptor undergoes conformational rearrangements that result in Hsp release and GR activation.^[10,16]^ Activated GR translocates into the nucleus, where it binds directly to its specific response element DNA sites and induces or represses the transcription of its target genes.^[10,16]^ The ligand-bound GR also cross-talks with other transcription factors, such as signal transducers, nuclear factor-kappa B, activator protein-1, and activators of transcription 5.^[16]^ Membrane-bound and cytosolic GR also act through nongenomic signaling pathways.^[16]^ Glucocorticoids are essential for life after birth and exert pleiotropic effects in different tissues.^[16]^ Some of these pleiotropic effects are achieved because ligand-bound GR also undergoes posttranslational modifications (e.g., phosphorylation, sumoylation, ubiquitination, acetylation, etc), which are tightly regulated through tissue-specific enzymes such as phosphatases or kinases.^[10]^ GR activity also depends on the composition of the recruited cofactor complex, which is dependent on cell-specific cofactor expression and cell context.^[10,17,18]^

### 3.2. GR as a driver of CRPC

**Figure 2.**
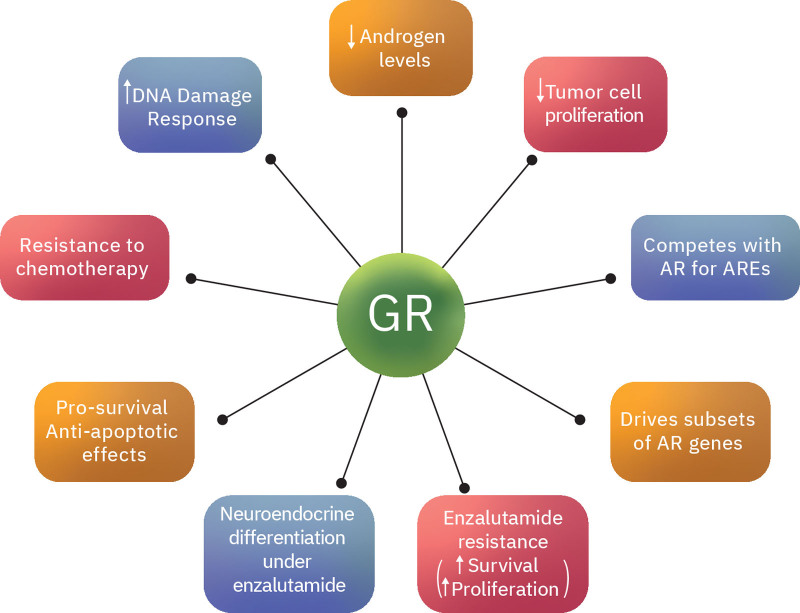
The role of GR in prostate cancer. Corticosteroids and GR have a complex role in prostate cancer. Although they can decrease proliferation in subsets of tumor cells, their prosurvival effects might render the cells resistant to chemotherapy and antiandrogen treatments, hence contributing to treatment failure. In the context of enzalutamide therapy, it has been shown that in subsets of primed cells, GR binds to AREs and activates critical AR genes. This results in GR-dependent tumor progression. AR = androgen receptor, ARE = androgen response element, GR = glucocorticoid receptor.

Given the close association between AR and GR, GR has been suggested to play a role in enzalutamide resistance in prostate cancer. GR and AR share overlapping transcriptomes and cistromes (Fig. 2). Arora et al^[7]^ developed enzalutamide-resistant tumors with high GR expression by adaptation to antiandrogen treatment in vitro. These cell lines were dependent on GR to maintain their drug-resistant phenotype. The addition of dexamethasone was sufficient to confer resistance to enzalutamide and drive proliferation, while inhibition of GR restored the sensitivity to treatment. Although patients treated with enzalutamide have low pretreatment GR expression levels, the percentage of GR-overexpressing cells within the tumor increases posttreatment, especially in patients who develop resistance and disease progression.^[7]^ The latter tumors showed uneven restoration of AR-target genes (Fig. 2). Arora et al^[7]^ showed that GR is the direct driver of a subset of critical AR-target genes in these tumors. Long-term treatment with abiraterone is also associated with GR overexpression.^[19]^ GR-mediated resistance to antiandrogen therapies is adaptive and reversible.^[20]^ In prostate cancer, GR expression is initially silenced by a combination of enhancer of zeste 2 polycomb repressive complex 2 subunit–mediated repression and AR binding at the GR locus (Fig. 1). However, in subsets of CRPC, GR expression is restored by reversion of both repressive signals.^[21]^ Interestingly, bromodomain and extraterminal domain bromodomain inhibitors impair the GR signaling axis in enzalutamide-resistant cells and resensitizes them to treatment.^[21]^

### 3.3. Antitumor effects of GR and the AR–GR interaction in prostate cancer

Overwhelming data suggest that GR signaling can drive tumor proliferation and even neuroendocrine differentiation under enzalutamide treatment.^[22,23]^ However, this is unlikely to be a major clinical problem for most other patients. Glucocorticoids have been used successfully for the treatment of prostate cancer for many years.^[24–26]^ In addition to alleviating the side effects of antiandrogen treatment, they can also suppress serum androgen levels.^[24,25,27]^ Their ability to decrease prostate-specific antigen levels and tumor volume is well documented.^[28,29]^ Experiments in preclinical models suggest that they also exert a direct inhibitory effect on prostate cancer cell proliferation and angiogenesis.^[30,31]^ GR-dependent growth under enhanced antiandrogen treatments occurs only in a subset of primed cells, and only after a few weeks of treatment.^[7]^ It has been shown that in the context of AR signaling, GR activation can slow the proliferation rate of prostate cancer cells^[32–34]^ (Fig. 2). GRs compete with AR for DNA-binding (the GR is also negatively regulated by active AR signaling).^[20,35]^ Although the 2 receptors share nearly identical DNA-binding specificities, the target genes overlap by <50% and execute unique functions in vivo.^[20,35]^ Different abilities of AR and GR to interact with relatively inaccessible chromatin result in divergent binding and corresponding gene regulation.^[36]^ There might also be subtle differences in the DNA-binding preferences between AR and GR. Shared binding sites display receptor-specific cofactor recruitment, enhancer activity, and histone modification changes.^[36]^ In addition, even when AR and GR upregulate the same transcriptional target gene, the effect might not be identical in magnitude.^[22]^ Hence, GR may be a much weaker activator of AR downstream signaling pathways.

### 3.4. Prosurvival effects of GR

Even at low levels, GR expression is very common in hormone therapy–naive patients, especially at metastatic sites.^[19]^ GR expression levels are not correlated with markers of aggressive phenotypes, such as prostate-specific antigen levels and tumor doubling time, etc.^[20]^ Frequently, GR overexpression after treatment is positively associated with sensitivity to antiandrogen therapies and signifies a regressing rather than a progressing tumor.^[20]^ Apart from long-term antiandrogen therapy, chemotherapy or radiation therapy also leads to GR overexpression.^[37,38]^ Besides being able to drive tumor proliferation, GR is a key player in prostate cancer cell survival.^[22,39]^ Stimulation of GR activity can rescue prostate cancer cells from enzalutamide-induced death.^[22]^ In vitro and in vivo experiments have shown that dexamethasone administration undermines the antitumor effects of paclitaxel and docetaxel, giving rise to chemotherapy-resistance.^[19,37]^ It can also confer resistance to radiation therapy.^[38]^ GR activation in prostate cancer can increase prosurvival gene expression.^[7,22,39,40]^ This effect is partly mediated by modulation of the antiapoptotic B-cell lymphoma 2/B-cell lymphoma-extra large axis.^[37]^ It can also affect adenosine monophosphate–activated protein kinase regulation of autophagy.^[39]^ Interestingly, GR activation results in decreased miR-99a/100 expression levels in prostate cancer stem-like cells.^[41]^ This promotes the regulation of DNA damage response via enhanced nuclear accumulation of switch/sucrose nonfermentable (SWI/SNF)-related, matrix-associated, actin-dependent regulator of chromatin, subfamily A, member 4 and SWI/SNF-related, matrix-associated, actin-dependent regulator of chromatin, subfamily D, member 1 (i.e., SWI/SNF chromatin remodeling factors) in DNA break points.^[41]^ In addition, GR activation exerts prosurvival and antiapoptotic activity by increasing the expression of serum and glucocorticoid-regulated kinase-1.^[22,42]^ Serum and glucocorticoid-regulated kinase-1 hinders Foxo3a-induced cell cycle arrest and apoptosis.^[43,44]^

### 3.5. Common protumorigenic effects of GR

**Figure 3.**
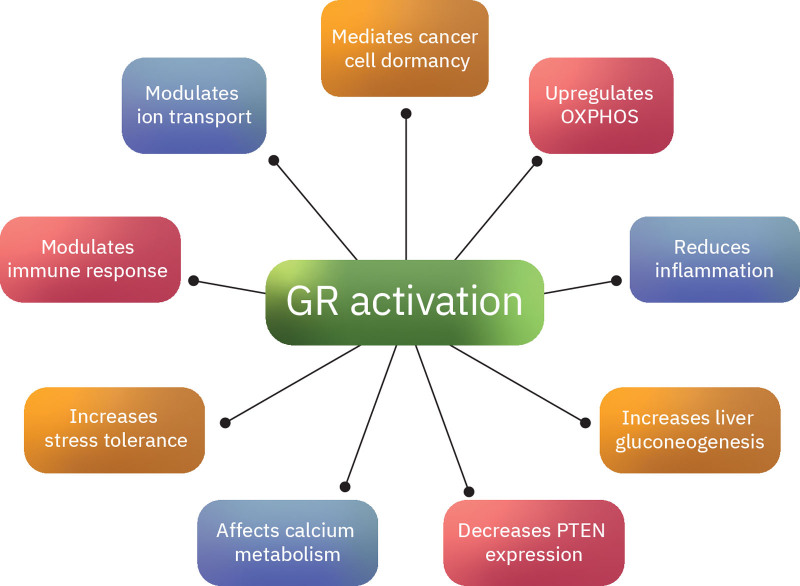
Pleiotropic effects of GR activation. Glucocorticoids modulate a variety of genes, including genes encoding chemokines and cytokines, enzymes, receptors, inhibitory proteins, and adhesion molecules. Many of the effects of GR activation are directly or indirectly related to cancer. These effects include ion transport modulation, immune response regulation, decreased PTEN expression, calcium metabolism regulation, inflammation reduction, etc. GR = glucocorticoid receptor, OXPHOS = oxidative phosphorylation, PTEN = phosphatase and tensin homolog.

GRs are present in almost all nucleated cells and have several mechanisms of action at the cellular level. Apart from their role in CRPC, glucocorticoids can also contribute to treatment failure in many other cancer types.^[45,46]^ Understanding the tumorigenic effects of GR activation in other tumor types might provide useful insights and generate interesting hypotheses regarding the effects of GR in prostate cancer. For example, in multiple cancer cell types, the GR signaling axis mediates cancer cell dormancy (Fig. 3). In particular, the tumors undergo cell cycle arrest through cyclin-dependent kinase inhibitor 1C and forkhead box O1/insulin receptor substrate 2–orchestrated reprogramming of signaling.^[47]^ The resulting quiescence slows the tumor proliferation rate. However, quiescent cancer cells can escape conventional antineoplastic treatments.^[48–51]^ They also minimize their metabolic rates and energy needs.^[52]^ Reactivation of these cells can eventually lead to tumor relapse and disease progression. Moreover, GR upregulates oxidative phosphorylation as a result of nuclear action on genes encoding mitochondrial transcription factors.^[53–55]^ In several tissues, GR activation leads to the translocation of the receptor into the mitochondria.^[55–58]^ Six mitochondrial genome sequences show strong similarity with glucocorticoid response elements, suggesting a direct action of GR on mitochondrial transcription.^[55,56,58^. It is known that GR binds to mitochondrial DNA, mainly in the regulatory D-loop region. Among the genes induced include the mitochondrially encoded oxidative phosphorylation genes *ND1*, *ND2, ND3, ND4, Cyt b, ATP6, ATP8, and COX1*. As a result, GR acts as a regulator that can increase OXPHOS and mitochondrial adenosine triphosphate production when the cells need it.^[55]^ It is known that the upregulation of OXPHOS can confer resistance to androgen ablation.^[59–61]^ These findings suggest that in prostate cancer under antiandrogen treatment, the mitochondrial GR translocation might have prosurvival effects and contribute to treatment failure. This is an interesting hypothesis because targeting GR translocation into the mitochondria would probably be a much safer strategy than targeting the (essential for life) GR directly.

### 3.6. “Landmark” effects of GR

Glucocorticoids modulate a variety of genes, including genes encoding chemokines and cytokines, enzymes, receptors, inhibitory proteins, and adhesion molecules (Fig. 3). Many of these effects are pleiotropic and cell/tissue specific.^[62,63]^ Overall, they upregulate anti-inflammatory proteins and downregulate proinflammatory proteins.^[62,64]^ In addition, they enhance the expression of enzymes involved in gluconeogenesis, particularly in the liver. This results in glucose synthesis from substrates such as amino acids and glycerol (from triglyceride breakdown). Glucocorticoids inhibit glucose uptake in muscle and adipose tissue, and stimulate lipolysis and fatty acid release in adipose tissue.^[65–68]^ GR activation can decrease phosphatase and tensin homolog (PTEN) expression. Increased PTEN levels are essential for the suppression of phosphoinositide 3-kinase–induced tumor growth.^[69]^ Glucocorticoids also play a role in calcium ion metabolism. Apart from decreasing renal calcium reabsorption, they decrease gastrointestinal calcium absorption and increase bone resorption by stimulating the expression of receptor activator of nuclear factor kappa B ligand. Hence, they enhance osteoclastogenesis and result in osteoporosis, a common side effect of chronic glucocorticoid treatment.^[70,71]^ This contrasts with the fact that prostate cancer bone metastases are mostly osteoblastic.^[72]^ Glucocorticoids are also key players in the restoration of homeostasis after exposure to stress. They modulate critical physiological responses, such as glycogenolysis, immune responses, and ion transport. Glucocorticoid secretion is a classic response to stress.^[73–75]^ Glucocorticoids are also critical for normal brain development.^[76,77]^ Moreover, by affecting Na+/K+/ATPase and nutrient transporters, they promote the development of a functional gastrointestinal system.^[78,79]^ In addition, they exert powerful effects on cellular excitability in neurons and neuroendocrine cells by regulating calcium-activated potassium channels.^[80]^

## 4. Conclusion

Glucocorticoids act through the GR and exert pleiotropic effects in different cell types. In prostate cancer, their role is somewhat complex. In the presence of active AR signaling, they can have an antitumor effect. However, under specific conditions, they contribute to resistance to antiandrogen therapies or even become the main driver of the disease. Identifying the full spectrum of GR activity will help inform the optimal use of glucocorticosteroids in prostate cancer. It will also determine the best strategies to target the protumorigenic effects of GR.

## Author contributions

M.S. conceptualized the review, reviewed the literature, drafted and critically reviewed the manuscript.

L.J.F. reviewed the literature and drafted the manuscript.

Both authors approved the final version of the manuscript.
